# Broken symmetry between RNA enantiomers in a crystal lattice

**DOI:** 10.1093/nar/gkab480

**Published:** 2021-06-09

**Authors:** Agnieszka Kiliszek, Leszek Błaszczyk, Magdalena Bejger, Wojciech Rypniewski

**Affiliations:** Institute of Bioorganic Chemistry, Polish Academy of Sciences, Noskowskiego 12/14, 61-704 Poznań, Poland; Institute of Bioorganic Chemistry, Polish Academy of Sciences, Noskowskiego 12/14, 61-704 Poznań, Poland; Institute of Bioorganic Chemistry, Polish Academy of Sciences, Noskowskiego 12/14, 61-704 Poznań, Poland; Institute of Bioorganic Chemistry, Polish Academy of Sciences, Noskowskiego 12/14, 61-704 Poznań, Poland

## Abstract

Explaining the origin of the homochirality of biological molecules requires a mechanism of disrupting the natural equilibrium between enantiomers and amplifying the initial imbalance to significant levels. Authors of existing models have sought an explanation in the parity-breaking weak nuclear force, in some selectively acting external factor, or in random fluctuations that subsequently became amplified by an autocatalytic process. We have obtained crystals in which l- and d-enantiomers of short RNA duplexes assemble in an asymmetric manner. These enantiomers make different lattice contacts and have different exposures to water and metal ions present in the crystal. Apparently, asymmetry between enantiomers can arise upon their mutual interactions and then propagate *via* crystallization. Asymmetric racemic compounds are worth considering as possible factors in symmetry breaking and enantioenrichment that took place in the early biosphere.

## INTRODUCTION

The chemical paradigm of symmetry between enantiomers stands in contrast to the predominance of l-amino acids and d-carbohydrates in living matter. Scientists attempting to explain the homochirality of biological molecules have considered possibilities that the asymmetry was prompted in the prebiotic world by a physical factor or arose by chance. One physical phenomenon that could ‘load the dice’ in favor of one enantiomer is the electroweak force active within molecules (1). However, its parity-breaking effect has not been measured and has been calculated to amount to 10^−11^ J mol^−1^, which corresponds to an excess of one molecule in 10^15^ (2). External factors that could tip the balance in favor of one enantiomer include a possible asymmetric lytic effect on enantiomers of circularly polarized light or spin-polarized particles (3,4). Hypotheses based on chance fluctuations include stereospecific autocatalytic reactions (5) or spontaneous resolution by crystallization (6). Either possibility requires that the tiny initial imbalance be amplified to an observable asymmetry. Autocatalytic reactions have been investigated extensively and recent findings indicate that no plausible phenomenon has yet been found ‘through which parity violating energy difference would be sufficiently ‘symmetry breaking’ to allow the subsequent asymmetric amplification that must occur to reach significant levels of enantioenrichment’ (7). Another approach to deracemization of oligonucleotides relies on template-directed synthesis. This model postulates the emergence of l- and d-enantiomeric libraries of ever increasing complexity, which would eventually become different from each other, simply because there are so many possible ways for them to become different. Finally, homochiral ‘winner sequences’ would emerge from one of the libraries (8).

Some of the proposed models rely on crystallization as a means by which any small imbalance at the molecular level could be amplified to the macroscopic scale. A few substances crystallizing as conglomerates, i.e., even mixtures of l- and d-crystals, could be nudged towards homochirality by mechanical agitation or heat. This phenomenon has been demonstrated for achiral molecules that give chiral crystals (9) and for chiral compounds that can undergo racemization when they are in solution (10). Another model involves crystals known as racemic compounds, in which the l- and d-enantiomers cocrystallize in a 1:1 proportion. Formation of such crystals removes equal amounts of each enantiomer from the solution, which should enhance any small initial imbalance in quantities of the enantiomers remaining in solution (11,12).

All the models of enantioenrichment involving crystallization have assumed structural symmetry between enantiomers, in accord with the chemical paradigm. A survey of ‘small molecule’ structures in the Cambridge Structural Database (13) reveals that racemic compounds generally cocrystallize in centrosymmetric space groups (over 90% of cases) or as separate l- and d-crystals (ca 6%). In approximately 1% of cases they crystallize as ‘kryptoracemates’ in chiral space groups, in which the enantiomers are not constrained by crystallographic symmetry (14). The conformations of the enantiomers in kryptoracemates are usually very similar, although differences have been noted. Kryptoracemates of small organic molecules are considered crystallographic oddities ‘because molecules that can be arranged around crystallographic inversion centers almost always are’ (15). The few racemic compounds of biological molecules, that have been examined to date, are centrosymmetric (16–18).

We have obtained crystals of a racemic compound consisting of l- and d-RNA duplexes in which the enantiomers pack in the crystal lattice in an asymmetric manner and show differences in their structures, crystal contacts and interactions with the solvent. The presented crystal structure is the first clear demonstration of asymmetry between the enantiomers of a biological molecule. We propose that this structural asymmetry could lead to a quantitative imbalance between the enantiomers.

## MATERIALS AND METHODS

The oligonucleotides CUGGGCGG and CCGCCUGG corresponding to domain E of *Thermus flavus* 5S rRNA (19) were synthesized in the d- and l-configurations and purified using the thin-layer chromatography (TLC) method on silica gel plates with ammonia/1-propanol/water solvent. The oligomers were eluted with water and lyophilized under vacuum via the CentriVap centrifugal vacuum concentrator (Labconco). RNA duplexes (CUGGGCGG)-(CCGCCUGG) of each enantiomeric form were annealed for 5 min in 95°C and cooled for 10 min on ice. Finally, oligomers were incubated at room temperature for 10 minutes and equal amounts of d- and l-duplexes were mixed to a final racemate concentration of 0.5 mM.

Crystals of the RNA racemate were grown at room temperature by the method of hanging drop/vapor diffusion in the presence of 0.2 M zinc acetate, 0.1 M cacodylate buffer, pH 6.5, and 18% (w/v) polyethylene glycol 8000, corresponding to solution 45 from Hampton Crystal Screen™. Each drop initially consisted of equal amounts of the RNA solution and the crystallization mixture. Needle-shaped crystals usually appeared within a few days. X-ray diffraction data were collected using synchrotron radiation at beamline P13, operated by EMBL Hamburg at the PETRA III storage ring (DESY, Hamburg, Germany) and at beamline MX14.1 at the BESSY II electron storage ring operated by Helmholtz-Zentrum Berlin (20). The crystals were mounted in a cryoloop from the crystallization drop and placed directly in a stream of nitrogen gas at 100 K. The diffraction data were recorded from multiple crystals on Pilatus 6M detectors and processed with XDS (21). The X-ray wavelength was set at 1.283 Å at the Zn absorption peak so that the anomalous scattering signal could be used to determine the absolute configuration of the molecules in the crystal. The structure was solved with Phaser (22), using as the starting models the d- and l-enantiomers of the previously determined structure (PDB code: 2GQ6) (18). The atomic models were refined using Refmac5 (23) and inspected and corrected using Coot (24). The solvent-accessible surface area was calculated with Areaimol (25) and the crystal contacts were analyzed with NCONT from the CCP4 program suite (26). Helical parameters were calculated using 3DNA (27).

Many crystals were tested and 17 gave processable data (Supplementary Table S1). The data sets were divided into two categories, according to how reliable they were for determining the absolute configuration of the crystal structure: (i) nine data sets had positive correlation between random half-sets of anomalous intensity differences (AnomalCorr) with values of the correlation coefficient between 0.12 and 0.47. The average anomalous difference expressed in units of its estimated standard deviation (SigAno) for those data sets was between 0.62 and 1.0. The resolution for those data ranged from 1.44 to 2.0 Å, and the *R*_merge_ statistics were in the range of 0.05–0.11. The maximum height of the anomalous density peaks at the Zn^2+^ ions in those structures ranged from 7 to 28 root-mean-square deviations (r.m.s.d.); (ii) Eight data sets did not have statistically significant AnomCorr (ranging from –0.17 to 0.03) and had SigAno in the range 0.51–0.63 but gave observable anomalous density at the Zn^2+^ ions. The resolution for those data sets ranged from 1.5 to 2.5 Å, and their *R*_merge_ was between 0.10 and 0.17. The maximum height of the anomalous peaks at the Zn^2+^ ions for those structures was in the range 5–17 r.m.s.d.. Thus, the anomalous signal in the second subset of data sets would probably be insufficient for solving the structure *ab initio*, but with the structure already known, the signal was useful for selecting the correct absolute configuration.

The anomalous density maps were calculated using as Fourier coefficients the anomalous amplitudes of the reflections, |F^+^-F^−^|, and phases calculated from the refined atomic coordinates and retarded by 90°. A second map was calculated for each crystal structure, with phases derived from symmetry-inverted coordinates. The absolute configuration of each crystal structure was determined by examining the two maps to determine which had significant density peaks corresponding to the Zn^2+^ ions. The alternative configuration always gave anomalous density maps with no significant peaks at the Zn^2+^ sites.

## RESULTS

**Figure 1.**
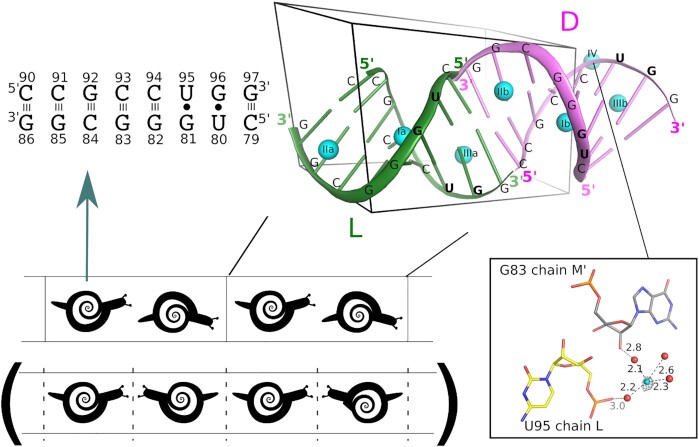
Nucleotide sequence, structure and crystal packing of the RNA duplexes. The sequence and base pairing (upper left). The l- and d-RNA duplexes (green and pink, respectively) in the crystal unit cell, with the bound Zn^2+^ ions (blue spheres) (upper right). Schematic representation of the asymmetric head-to-tail crystal packing of RNA duplexes compared to symmetric head-to-head/tail-to-tail packing (in brackets, below) (lower left). The unpaired Zn^2+^ ion (blue sphere) with its anomalous electron density is shown at the 4 σ level (gray contours); water molecules are shown as red spheres, and distances are given in angstroms (inset, lower right). The model and the electron density map in this and other figures are based on the data set 140-F4a (PDB code: 6ZPF).

**Figure 2.**
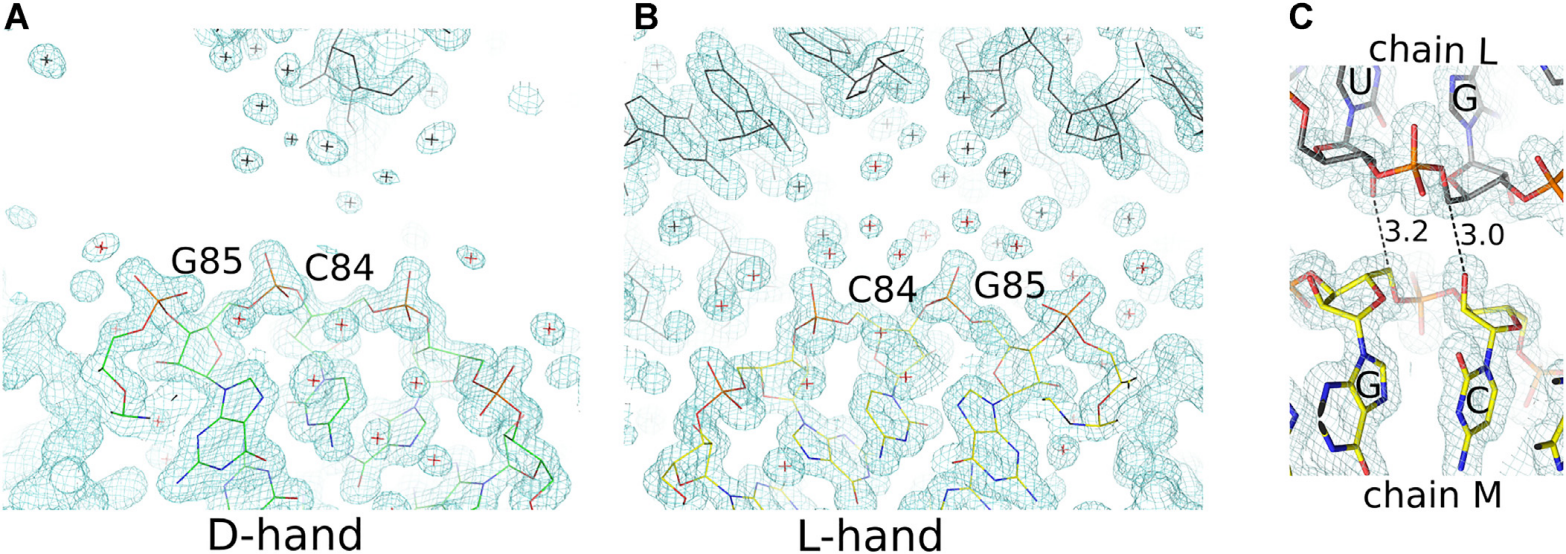
Asymmetry of crystal packing and solvent exposure (A, B) Corresponding parts of the l- and d-RNA duplexes. The 2*F*_o_–*F*_c_ electron density is shown at the 1σ level (blue contours), and ordered water molecules are marked with crosses. (C) Interactions between chains L and M that have no symmetric equivalents between chains K and N. Hydrogen bonds are shown between O2’ C84M and C5’ G96L and between C5’ G85M and O2’ U95L. Distances are shown in angstroms.

The crystals of d- and l-RNA duplexes (CUGGGCGG)-(CCGCCUGG) belong to space group P1 with the unit cell parameters *a* = 21.1 Å, *b* = 26.7 Å, *c* = 39.1 Å, *α* = 105.9°, *β* = 97.0°, and *γ* = 91.6°. The unit cell contains one d-RNA duplex and one l-RNA duplex stacking head-to-tail, arranged in the crystal lattice into parallel semi-infinite columns (Figure 1). In addition, the unit cell contains seven Zn^2+^ ions and 150–180 ordered water molecules, depending on the data set. The d-duplexes have the helical parameters characteristic of A-RNA, while the l-duplexes take values appropriately inverted for the other enantiomer (Supplementary Table S2). The two duplexes (whose chains have been labeled K:L and M:N) have a slightly higher average twist (35° for duplex K:L, 34° for M:N) than a typical A-RNA (32°). This result is due to the first U–G pair, which has a twist of 44–46°. The pair also has an elevated buckle (9°), and the roll of the UG/UG step is also high (15–16°). The C–G pairs show the standard Watson–Crick pairing, while the U–G pairs show the typical wobble (Supplementary Figure S1A, B). A comparison of the two RNA enantiomers, after an appropriate symmetry operation, reveals a root-mean-square difference in atomic coordinates of approximately 0.22–0.25 Å, with maximum differences of 0.5–0.75 Å, depending on the data set. The intermolecular crystal contacts of the two enantiomers are also different (Supplementary Table S3). When the two duplexes (K:L and M:N) are compared, chain K is more exposed to the solvent than its corresponding enantiomer, chain M, which makes more extensive contacts with neighboring RNA molecules (Figure 2A, B). The percentage ratio of the solvent-exposed surface area to the intermolecular crystal contact area is 72/28 for chain K and 57/43 for chain M. The situation is reversed for the other strands of the two duplexes: the ratios of the solvent-exposed intermolecular contact areas are 55/45 for chain L and 71/29 for chain N.

The crystal contacts between the RNA duplexes involve the ribose moieties, the phosphate groups and the N2 amine groups of guanine residues (Supplementary Table S3). The two duplexes make 14 such contacts, 12 of which form corresponding pairs for the two enantiomers, while two contacts are asymmetric. The unusual close interactions between O2’ C84 of chain M and C5’ G96, chain L (3.0 Å), and between C5’ G85, chain M, and O2’ U95, chain L (3.2 Å), have no matching contacts in chains K and N (Figure 2C). The two contacts resemble a ‘ribose zipper’ (28), linking two consecutive ribose moieties of two neighboring RNA chains. The resemblance is limited because the contacts in the present structure involve different enantiomers and instead of O2’–O2’ hydrogen bonds there are O2’-C5’ interactions. Hydrogen bonds involving methylene groups are not usual but close contacts involving C5’ have been seen before in nucleic acids (PDB code: 1JJ2) (29). The C5’ methylene group is relatively reactive and the C-H bonds can be labile (30,31). It seems that the C5’ atom can act as a hydrogen bond donor.

Asymmetry between the RNA enantiomers is also observed in their interactions with the Zn^2+^ ions. The crystal structure contains seven metal ions of which six form pairs bound in the major groove at corresponding sites at the l- and d-duplexes (Figure 1). The first pair of Zn^2+^ ions forms inner-sphere complexes with the N7 atom of residues G82 of chains K and M (Supplementary Figure S1C). The second pair forms octahedrally hydrated complexes interacting with residues G85 and G86 of chains K and M. The third pair of Zn^2+^ ions is located at residues G96 and G97 of chains L and N. The seventh Zn^2+^ ion binds *via* water molecules to the phosphate groups of residues C94 and U95 of chain L. In addition, one of the coordinating water molecules of the seventh Zn^2+^ ion bridges the U95 phosphate group with the O2’ hydroxyl group of residue G83 in strand M of a neighboring duplex (Figure 1, inset). This Zn^2+^ ion has no corresponding site at the other enantiomer, presumably because the crystal packing is different for chains L and N and there is no neighboring RNA duplex to stabilize Zn^2+^ at the corresponding site in chain N.

Both RNA duplexes show a degree of disorder in the sense that a fraction of 0.1–0.2 of the duplexes has an inverted orientation. When the predominant conformation is modeled and refined, the minor conformation manifests itself as peaks on the difference electron density map in places where the nucleotide sequence departs from being palindromic, i.e. at the two central C-G pairs and at the flanks of the duplex where the nucleobases U and C become superimposed. The Zn^2+^ ions associated with the central GG step in each RNA duplex also have minor sites that are superposed on the water structure near C93 of the major conformation. A comparison of the occupancy factors of the Zn^2+^ ions indicates that the disorder appears to be lower at the RNA duplex K:L than at M:N. Various degrees of disorder are also observed in the water surrounding the RNA. Ordered water molecules predominate in areas of contact between the RNA molecules, whereas the larger voids between the molecules tend to be filled with disordered solvent. Consequently, the RNA chains more exposed to the solvent are more exposed to disordered solvent.

The absolute configuration of the structure was determined for each analyzed crystal using the anomalous dispersion of X-rays from the Zn^2+^ ions. Of the nine crystals that gave diffraction data containing statistically significant anomalous signals, seven crystals had the K:L RNA duplex in the d-configuration, and the M:N duplex was in the l-configuration (Supplementary Table S1). The remaining two crystals had the inverted configuration l-(K:L)/d-(M:N). Of the eight crystals that gave no statistically significant anomalous signal but gave anomalous peaks at the Zn^2+^ ions, four crystals were d-(K:L)/l-(M:N), and four were l-(K:L)/d-(M:N).

## DISCUSSION

The main conclusion from analyzing this crystal structure is that the head-to-tail packing of the d- and l-RNA duplexes precludes symmetry between them (Figure 1). The asymmetry is evident in the contacts between the RNA molecules, their exposure to the solvent, the structure of the solvent and of the different enantiomers. It is common for RNA or DNA oligomers to stack end-to-end in the crystal lattice, and for nonpalindromic sequences there are two distinct stacking possibilities: symmetric head-to-head/tail-to-tail or asymmetric head-to-tail. There is no *a priori* reason to rule out the possibility that the molecules could preferentially stack head-to-tail and then assemble into an asymmetric crystal lattice. However, it is unusual for enantiomers to pack asymmetrically. This case needs to be distinguished from the common case of multiple molecules occupying general positions in the asymmetric unit, because the common case applies to molecules that are chemically identical. Enantiomers, on the other hand, usually form symmetric lattices. The exceptions are the kryptoracemates known from small-molecule crystallography, amounting to approximately 1% of crystalline racemates found in the CSD (13).

A careful examination of the electron density maps reveals a degree of static disorder indicating a balance between the head-to-head and head-to-tail packing of the molecules, with predominance of the asymmetric head-to-tail association. Another crystal form of this racemic compound had head-to-head and head-to-tail stacking balanced evenly, which resulted in a centrosymmetric structure (18).

The asymmetry is unlikely to be caused by the presence of a chiral contaminant. There are no chiral components in the crystallization solution and even if one suspected an unknown contaminant, it should be noted that the crystallization can go ‘either way’, giving crystals of opposite handedness under similar conditions. The two forms are symmetric to each other but asymmetric each in itself. The ratio of the d-(K:L)/l-(M:N) to l-(K:L)/d-(M:N) crystal structures, of 7 to 2 for the best data sets and 11 to 6 overall, does not indicate a clear bias. Thus, there is probably no evidence in these results of the parity-breaking weak nuclear force.

The novelty of these results is the asymmetry in a single crystal between the biologically relevant enantiomers. With regard to the question of the origin of biomolecular homochirality, it should be noted that the asymmetric racemic compounds seem to fulfill both basic requirements for ‘deracemisation’: enantioselectivity and enantioenrichment. The mirror symmetry between the enantiomers can apparently be broken when they interact with each other. Crystallization then amplifies this instantaneous asymmetry from the molecular level to the macro scale. In the crystal lattice, the different enantiomers remain trapped in different states, having different contacts with the neighboring molecules and different exposure to the solvent present in the lattice which, in addition to water, contains metal ions. In the presented structure, one enantiomer interacts with three zinc cations, while the other interacts with four zinc cations. This should have an effect on the relative lifetimes of the enantiomers. In consequence, different amounts of l- and d-molecules should remain after a time. In large volumes, the effects of crystalline asymmetric racemic compounds and their mirror images (in this study: d-(K:L)/l-(M:N) and l-(K:L)/d-(M:N)) will tend to average out, but in smaller volumes a significant lasting disproportion becomes more likely (consider a drop with only one crystal). It should also be noted that this model requires no initial imbalance between the enantiomers. Even a perfect balance in solution can turn spontaneously into a clear non-equivalence in the crystal lattice.

Researchers have been looking for ways by which fluctuations at the molecular level could have been amplified to the macroscopic level (7) and crystallization has been considered as a possible means to bridge the two scales. Asymmetric racemic compounds are novel balance-tipping elements to be considered. Interestingly, asymmetric racemic compounds are demonstrated here for RNA—a biological molecule hypothesized to have predominated in the early biosphere.

## DATA AVAILABILITY

Atomic coordinates and structure factors for the reported crystal structures have been deposited with the Protein Data Bank under accession numbers 6ZQ9, 6ZR1, 6ZPF, 6ZRL, 6ZRS, 6ZX8, 7A9L, 7A9N, 7A9O, 6ZW3, 6ZWU, 6ZX5, 7A9P, 7A9Q, 7A9R, 7A9S, 7A9T. Diffraction images have been deposited in Macromolecular Xtallography Raw Data Repository (MX-RDR, mxrdr.icm.edu.pl). The DOI addresses of the raw data sets are given in Supplementary Table S1.

## Supplementary Material

gkab480_Supplemental_File
