# Supplementary material for: Broken symmetry between RNA enantiomers in a crystal lattice
Source: Nucleic Acids Res. 2021 Jun 9;49(21):12535–9. doi: 10.1093/nar/gkab480 (PMC8643679; doi:10.1093/nar/gkab480)
Supplement: gkab480_Supplemental_File [file gkab480_supplemental_file.docx]

**SUPPLEMENTARY DATA**

**to**

**Broken symmetry between RNA enantiomers in a crystal lattice**

Agnieszka Kiliszek, Leszek Błaszczyk, Magdalena Bejger, Wojciech Rypniewski^*^

Institute of Bioorganic Chemistry, Polish Academy of Sciences, Noskowskiego 12/14, 61-704 Poznań, Poland.


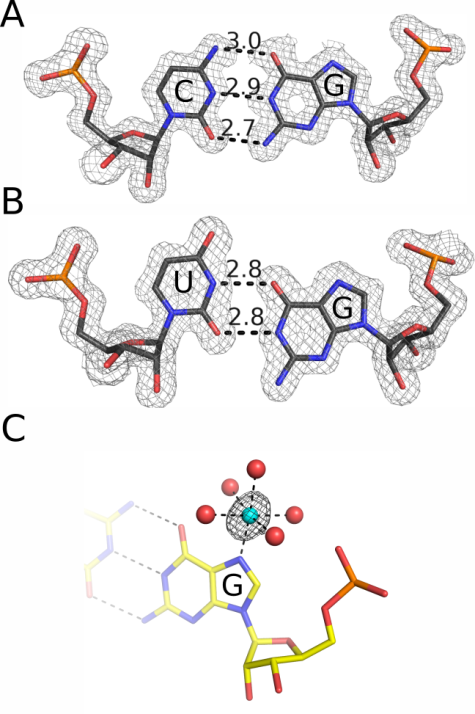


**Supplementary Figure S1. Base pairing and interactions with Zn^2+^** (**A**, **B**) C-G and U-G base pairs with the *2F_o_-F_c_* electron density map contoured at the 1σ level (gray contours). Distances of hydrogen bonds are marked in angstroms. (**C**) A hydrated Zn^2+^ ion (blue sphere) forming an inner complex with the N7 atom of residues G82 of chain K, with its anomalous electron density contoured at the 4 σ level (gray contours). The water molecules are shown as red spheres.

**Supplementary Table S1**. Summary of X-ray diffraction data.

| Crystal name | PDB code | Resoln. [Å] | Wave-length [Å] | R_merge_^#^ | R^§^/R_free_^$^ | ‘hand’^£^ | AnomalCorr | SigAno | Anomalous peak height [σ]^¥^ | | | | | | | X-ray facility/date | DOI address of diffraction images^*^ |
| --- | --- | --- | --- | --- | --- | --- | --- | --- | --- | --- | --- | --- | --- | --- | --- | --- | --- |
|  |  |  |  |  |  |  |  |  | ZN1 | ZN2 | ZN3 | ZN4 | ZN7 | ZN8 | ZN10 |  |  |
| 136-E1a | 6ZQ9 | 1.50 | 1.283 | 0.079 (0.967) | 0.201/0.250 | + | 0.20 | 0.75 | 16.5 | 16.1 | 7.0 | 12.1 | 15.6 | 9.4 | 8.7 | EMBL/01-12-2018 | 10.18150/9ACEMY |
| 140-F2a | 6ZR1 | 1.53 | 1.283 | 0.086 (0.557) | 0.203/0.253 | + | 0.21 | 0.80 | 18.3 | 15.3 | 9.1 | 9.8 | 12.1 | 10.8 | 7.5 | EMBL/01-12-2018 | 10.18150/C7CNYT |
| 140-F4a | 6ZPF | 1.44 | 1.283 | 0.052 (0.469) | 0.201/0.251 | + | 0.35 | 0.88 | 24.1 | 21.0 | 9.5 | 6.4 | 19.8 | 13.9 | 6.3 | EMBL/01-12-2018 | 10.18150/JV8T9Y |
| 140-F6b | 6ZRL | 1.53 | 1.283 | 0.107 (0.731) | 0.213/0.275 | - | 0.25 | 0.84 | 15.8 | 18.8 | 9.4 | 9.4 | 13.0 | 11.5 | 8.1 | EMBL/01-12-2018 | 10.18150/SMC7KS |
| 140-F6c | 6ZRS | 1.52 | 1.283 | 0.069 (0.566) | 0.187/0.235 | + | 0.24 | 0.82 | 18.2 | 17.3 | 9.5 | 11.8 | 15.0 | 10.5 | 7.4 | EMBL/01-12-2018 | 10.18150/BELVHE |
| 143-A7d | 6ZX8 | 1.98 | 1.283 | 0.099 (0.479) | 0.173/0.259 | + | 0.12 | 0.62 | 6.8 | 7.6 | 4.9 | 5.5 | 9.5 | 4.8 | 2.2 | BESSY/02-02-2019 | 10.18150/P4L74Z |
| 143-A2c | 7A9L | 1.98 | 1.283 | 0.103 (0.407) | 0.174/0.250 | - | 0.17 | 0.65 | 7.1 | 6.7 | 3.9 | 3.5 | 6.2 | 4.1 | 3.1 | BESSY/02-02-2019 | 10.18150/Q4UKIN |
| 136-E1b | 7A9N | 1.60 | 1.283 | 0.072 (0.472) | 0.180/0.224 | + | 0.45 | 1.00 | 27.5 | 22.4 | 12.8 | 6.6 | 5.9 | 4.9 | - | EMBL/01-05-2019 | 10.18150/IEYXA5 |
| 143-A11b | 7A9O | 1.60 | 1.283 | 0.089 (0.510) | 0.191/0.228 | + | 0.47 | 0.96 | 19.3 | 16.4 | 9.0 | 11.2 | 10.5 | 9.2 | 7.5 | EMBL/01-05-2019 | 10.18150/QFD8JM |
| 140-F4b | 6ZW3 | 1.50 | 1.283 | 0.168 (1.061) | 0.213/0.272 | - | -0.02 | 0.57 | 16.5 | 15.8 | 8.0 | 4.2 | 13.1 | 9.0 | 4.1 | EMBL/01-12-2018 | 10.18150/KWD3XQ |
| 140-F4c | 6ZWU | 1.53 | 1.283 | 0.135 (1.187) | 0.198/0.252 | - | -0.05 | 0.62 | 13.5 | 13.2 | 5.7 | 6.9 | 12.8 | 9.2 | 5.1 | EMBL/01-12-2018 | 10.18150/GHURCS |
| 141-A6 | 6ZX5 | 1.52 | 1.283 | 0.164 (1.526) | 0.211/0.281 | + | -0.11 | 0.53 | 10.3 | 9.2 | 5.2 | 8.1 | 9.8 | 5.3 | 4.1 | EMBL/01-12-2018 | 10.18150/FVWGG8 |
| 143-D7b | 7A9P | 1.99 | 1.277 | 0.100 (0.256) | 0.235/0.326 | - | -0.17 | 0.61 | 4.7 | 7.0 | 2.7 | 2.8 | 5.4 | 5.8 | - | BESSY/02-02-2019 | 10.18150/HLJBOJ |
| 142-C1b | 7A9Q | 2.19 | 1.283 | 0.131 (0.452) | 0.164/0.232 | + | 0.03 | 0.56 | 7.4 | 7.3 | 3.1 | 5.2 | 7.0 | 3.8 | 2.8 | BESSY/02-02-2019 | 10.18150/4ZIRX1 |
| 142-C1c | 7A9R | 2.46 | 1.283 | 0.121 (0.352) | 0.178/0.284 | - | -0.10 | 0.54 | 5.3 | 4.8 | 2.6 | 3.1 | 4.1 | 3.8 | - | BESSY/02-02-2019 | 10.18150/EBSJ8O |
| 142-C1d | 7A9S | 2.30 | 1.283 | 0.133 (0.474) | 0.169/0.274 | + | -0.13 | 0.51 | 7.4 | 8.7 | 4.2 | 5.9 | 5.2 | 4.4 | 2.8 | BESSY/02-02-2019 | 10.18150/DK6OUQ |
| 141-A6b | 7A9T | 1.70 | 1.283 | 0.100 (0.350) | 0.188/0.248 | + | -0.02 | 0.63 | 11.8 | 9.9 | 4.8 | 5.7 | 5.3 | 5.1 | 3.8 | EMBL/01-05-2019 | 10.18150/W45YHJ |

^#^R_merge_ = Σ_hkl_Σ_i_|I_i_(hkl) - <I(hkl)>|/ Σ_hkl_Σ_i_I_i_(hkl), where I_i_(hkl) is the integrated intensity of a given reflection and <I(hkl)> is the mean intensity of multiple corresponding symmetry-related reflections. The values in parentheses are for the highest resolution shell.

^§^R = Σ_hkl_||F_obs_|-|F_calc_||/ Σ_hkl_ |F_obs_|, where F_obs_ and F_calc_ are the observed and calculated structure factors, respectively.

^$^R_free_ is R calculated using a randomly chosen subset of reflections excluded from the refinement.

^£^ ‘+’ indicates enantiomers d-(K:L)/l-(M:N) and ‘-‘ indicates l-(K:L)/d-(M:N)

^¥^ The height of peaks on the anomalous map, in terms of r.m.s.d, corresponding to the Zn^+2^ sites, used to determine the ‘hand’ of the X-ray structures. The anomalous maps calculated for the opposite ‘handedness’ showed no significant peaks.

^*^ The diffraction images are available at Macromolecular Xtallography Raw Data Repository (MX-RDR): mxrdr.icm.edu.pl

**Supplementary Table S2**. Helical parameters (based on crystal 140-F4a, PDB code 6ZPF) calculated using 3DNA {Olson, 2001 #76}.

*K+L duplex*

Local base-pair parameters

| B.p. | Shear  [Å] | Stretch  [Å] | Stagger  [Å] | Buckle  [°] | Propllr.  [°] | Openg.  [°] | Displ.  [Å] | Angle  [°] | Twist  [°] | Rise  [Å] |
| --- | --- | --- | --- | --- | --- | --- | --- | --- | --- | --- |
| 1 c-g | -0.0 | -0.1 | 0.2 | 4.5 | -7.0 | -2.0 | 5.9 | 13.2 | 28.0 | 3.0 |
| 2 u-g | 2.4 | -0.6 | -0.1 | 9.6 | -19.7 | -0.9 | 5.3 | 9.7 | 44.4 | 2.6 |
| 3 g-u | -2.3 | -0.7 | 0.2 | -1.0 | -9.3 | -3.1 | 5.8 | 11.1 | 27.2 | 2.9 |
| 4 g-c | -0.3 | -0.2 | 0.4 | 4.7 | -15.3 | 0.4 | 5.7 | 12.3 | 38.1 | 2.4 |
| 5 g-c | -0.1 | -0.3 | 0.1 | -2.7 | -14.8 | -3.9 | 5.8 | 11.6 | 32.4 | 2.6 |
| 6 c-g | 0.2 | -0.2 | 0.1 | -1.1 | -15.8 | 1.0 | 5.8 | 10.4 | 36.3 | 2.7 |
| 7 g-c | -0.3 | -0.3 | -0.1 | -10.4 | -16.8 | -3.0 | 5.9 | 10.5 | 32.2 | 2.9 |
| 8 g-c | -0.2 | -0.1 | 0.1 | -7.8 | -8.0 | -3.3 | 5.9 | 15.0 |  |  |
| ave. | -0.1 | -0.3 | 0.1 | -0.5 | -13.3 | -1.9 | 5.8 | 11.7 | 34.1 | 2.7 |
| s.d. | 1.3 | 0.2 | 0.1 | 6.7 | 4.6 | 1.8 | 0.2 | 1.6 | 5.6 | 0.2 |

Local base-pair step parameters

| Step | Shift  [Å] | Slide  [Å] | Rise  [Å] | Tilt  [°] | Roll  [°] | Twist  [°] |
| --- | --- | --- | --- | --- | --- | --- |
| 1 cu/gg | 0.0 | -1.1 | 3.2 | 0.9 | 7.2 | 39.8 |
| 2 ug/ug | -0.9 | -2.3 | 3.1 | -5.0 | 15.1 | 18.0 |
| 3 gg/cu | 0.1 | -1.0 | 3.2 | 0.2 | 2.3 | 37.4 |
| 4 gg/cc | -0.3 | -1.2 | 3.3 | 0.1 | 7.3 | 38.3 |
| 5 gc/gc | 0.4 | -1.3 | 3.2 | 0.1 | 2.0 | 33.3 |
| 6 cg/cg | 0.1 | -1.7 | 3.3 | 3.6 | 14.8 | 32.3 |
| 7 gg/cc | 0.3 | -1.5 | 3.2 | 2.0 | 7.7 | 31.4 |
| ave. | -0.0 | -1.4 | 3.2 | 0.3 | 8.1 | 32.9 |
| s.d. | 0.4 | 0.4 | 0.1 | 2.6 | 5.3 | 7.3 |

*M+N duplex*

Local base-pair parameters

| B.p. | Shear  [Å] | Stretch  [Å] | Stagger  [Å] | Buckle  [°] | Propllr.  [°] | Openg.  [°] | Displ.  [Å] | Angle  [°] | Twist  [°] | Rise  [Å] |
| --- | --- | --- | --- | --- | --- | --- | --- | --- | --- | --- |
| 1 c-g | 0.0 | -0.1 | 0.2 | 5.0 | 8.0 | 2.6 | 6.0 | 13.8 | -25.3 | 3.0 |
| 2 u-g | -2.5 | -0.6 | 0.0 | 8.7 | 18.0 | -2.1 | 5.6 | 9.8 | -45.4 | 2.6 |
| 3 g-u | 2.1 | -0.6 | 0.1 | -2.5 | 12.6 | 2.2 | 5.6 | 10.8 | -27.6 | 2.8 |
| 4 g-c | 0.1 | -0.1 | 0.3 | 2.5 | 17.4 | 0.5 | 5.7 | 12.1 | -37.0 | 2.5 |
| 5 g-c | 0.3 | -0.2 | 0.0 | -5.6 | 16.1 | 1.8 | 5.9 | 11.7 | -31.2 | 2.9 |
| 6 c-g | -0.5 | -0.2 | 0.1 | 0.0 | 13.2 | -1.6 | 5.6 | 11.9 | -37.9 | 2.6 |
| 7 g-c | 0.3 | -0.3 | -0.1 | -9.4 | 19.8 | 3.3 | 5.5 | 11.0 | -33.0 | 2.9 |
| 8 g-c | 0.2 | -0.2 | 0.1 | -7.0 | 9.4 | 3.1 | 5.7 | 14.5 |  |  |
| ave. | 0.0 | -0.3 | 0.1 | -1.0 | 14.3 | 1.2 | 5.7 | 12.0 | -33.9 | 2.8 |
| s.d. | 1.3 | 0.2 | 0.1 | 6.3 | 4.2 | 2.1 | 0.2 | 1.5 | 6.3 | 0.2 |

Local base-pair step parameters

| Step | Shift  [Å] | Slide  [Å] | Rise  [Å] | Tilt  [°] | Roll  [°] | Twist  [°] |
| --- | --- | --- | --- | --- | --- | --- |
| 1 cu/gg | -0.0 | -1.2 | 3.2 | -0.6 | -7.9 | -38.3 |
| 2 ug/ug | 0.6 | -2.2 | 3.1 | -2.7 | -15.7 | -19.6 |
| 3 gg/cu | 0.1 | -1.1 | 3.2 | 0.1 | -2.5 | -37.2 |
| 4 gg/cc | 0.2 | -1.2 | 3.3 | 0.7 | -8.1 | -35.3 |
| 5 gc/gc | -0.6 | -1.0 | 3.1 | -0.1 | -4.3 | -34.5 |
| 6 cg/cg | -0.0 | -1.5 | 3.4 | 3.7 | -13.0 | -32.4 |
| 7 gg/cc | -0.3 | -1.2 | 3.2 | 1.1 | -7.7 | -32.0 |
| ave. | 0.0 | -1.3 | 3.2 | 0.5 | -8.4 | -32.7 |
| s.d. | 0.4 | 0.4 | 0.1 | 1.9 | 4.6 | 6.2 |

**Supplementary Table S3**. Intermolecular contacts in the crystal structure of d-(K:L)/l-(M:N) RNA duplexes (based on crystal 140-F4a, PDB code 6ZPF), other than base-pairing and base-stacking contacts. The distance range is 2.15-3.25 Å, W stands for water molecule, symmetry-related molecules are marked with *, direct contacts between neighboring RNA duplexes are underscored, lattice contacts with no symmetric counterparts are highlighted in color.

| **Residue** | **Atom** | **Chain K** | **Chain M** | **Residue** | **Atom** | **Chain L** | **Chain N** |
| --- | --- | --- | --- | --- | --- | --- | --- |
|  |  |  |  |  |  |  |  |
| **C79** | O5’ |  | W141 | **C90** | O5’ | O3’ G97N, W143 | O3’ G97L*, W135, W89*, W91* |
|  | O4’ | W38 |  |  | O2’ | W93, W182 | W151* |
|  | O2’ | W79, W98 | W59 |  | N4 |  | W107 |
|  | O2 | W194 |  |  | O2 | W160, W182 | W147*, W151* |
|  |  |  |  |  |  |  |  |
| **U80** | OP1 | W69 |  | **C91** | OP1 | W51, W143 | W52, W135 |
|  | O4’ | W73 | W59 |  | OP2 | W175, W176 | W99 |
|  | O3’ | W56 | W190 |  | O2’ | W56* | W32* |
|  | O2’ | W44, W56 | W32, W50 |  | N4 | W110, W165 | W144 |
|  | O2 | W44 | W50 |  | O2 | W20 | W11 |
|  | O4 | W75,W189 |  |  |  |  |  |
|  |  |  |  |  |  |  |  |
| **G81** | OP1 | W22, W28 | W5, W113 | **G92** | OP1 | W4 | W7, W65 |
|  | OP2 | W145 | W80, W190 |  | OP2 | W103 |  |
|  | O4’ | O4’ G92L* | O4’ G92N* |  | O5’ | W4 |  |
|  | O3’ | W20* | W11* |  | O4’ | O4’ G81K* | O4’ G81M* |
|  | O2’ | N2 G85K*, W43*, W106* | N2 G85M* (3.5Å), W155 |  | O2’ | N2 G96L*, W139 | N2 G96N* |
|  | N7 |  | W113 |  | N7 | W157 | W65, W136 |
|  | N3 | W139* |  |  | O6 | W165 | W84, W136 |
|  | O6 | W75 |  |  | N3 | W106 | W155* |
|  | N2 | W31, W170 | W101, W102 |  |  |  |  |
|  | N3 | W139* |  |  |  |  |  |
|  |  |  |  |  |  |  |  |
| **G82** | OP1 | W22, W118 | W5, W64, W80 | **C93** | OP1 | W4, W29 | W1, W7 |
|  | OP2 | O2’ G86K*, W140* | O2’ G86M*, W105 |  | OP2 | O2’ G97L*, W18 | O2’ G97N*, W25 |
|  | O3’ | W49* | W15* |  | O3’ | W21* | W45* |
|  | O2’ | W39, W49* | W14*, W15*, W166* |  | O2’ | W16, W21* | W58 |
|  | N7 | ZN1, W2, W22, W117, W118 | ZN2, W5, W27, W64, W112 |  | N4 | W72, W162 | W163 |
|  | O6 | W117 | W112 |  | O2 | W96 | W58 |
|  | N2 | W67 | W76 |  |  |  |  |
|  |  |  |  |  |  |  |  |
| **G83** | OP1 | W37, W55, W187 | W33, W63, W88 | **C94** | OP1 | W48, W74 | W40, W61* |
|  | OP2 | W47, W66 | W134, W9* |  | OP2 | W14, W12* | W23, W94 |
|  | O5’ | W187 |  |  | O5’ |  | W40 |
|  | O4’ | W39 |  |  | O3’ |  | W57 |
|  | O2’ |  | W12, W19, W46 |  | O2’ | W9*, W26* | W57 |
|  | N7 | W37 | W33 |  | N4 | W142 | W127 |
|  | O6 | W2 | W27 |  | O2 | W41 | W137 |
|  | N3 | W41 | W19 |  |  |  |  |
|  |  |  |  |  |  |  |  |
| **C84** | OP1 | W149, W187 | W63, W179 | **U95** | OP1 | W48, W83, W100 | W40 |
|  | OP2 | W82 | W26, W146, W16* |  | OP2 | W14, W46* |  |
|  | O5’ | W149 | W179 |  | O4’ | W90* |  |
|  | O2’ |  | C5’ G96L* (3.0Å), W17 |  | O2’ | C5’ G85M* (3.2Å), W31, W90* | W102 |
|  | O2 | W96 | W17 |  | O4 | W77, W129, W189 | W30 |
|  | N4 | W10 |  |  |  |  |  |
|  |  |  |  |  |  |  |  |
| **G85** | OP1 | W121, W149 | W70, W179 | **G96** | OP1 | W79, W186 | W85, W133 |
|  | OP2 |  | W8, W111, W31* |  | OP2 | W13, W192, W17* |  |
|  | C5’ |  | O2’ U95L* (3.2Å) |  | O5’ | W79 |  |
|  | O4’ |  | W17 |  | C5’ | O2’ C84M* (3.0Å) |  |
|  | O3’ |  | W90 |  | O4’ |  | W102 |
|  | O2’ | W43, W49, W171* | W3, W15, W166 |  | O2’ | W21, W8* | W45, W178 |
|  | N7 | W120, W159 | W183 |  | N7 | W129, W138 |  |
|  | O6 | W122 | W156 |  | O6 |  | W188 |
|  | N3 | W43 | W3 |  | N2 | O2’ G92L*, W44 | O2’ G92N*, W50 |
|  | N2 | O2’ G81K* | O2’ G81M* (3.5Å) |  |  |  |  |
|  |  |  |  |  |  |  |  |
| **G86** | OP1 | W121 | W196, W38* | **G97** | OP1 | W79, W97 | W85, W86 |
|  | OP2 | W42, W47* | W24, W90 |  | OP2 | W60, W71, W12* | W78, W87 W23* |
|  | O4’ | W154* |  |  | O3’ | O5’ C90N*, W89, W91 | O5’ C90L*, W61 |
|  | O3’ | W141, W185, W55* | W184, W88* |  | O2’ | OP2 C93L*, W74* | OP2 C93N*, W61 |
|  | O2’ | OP2 G82K*, W197, W55*, | OP2 G82M*, W88*, W105* |  | N7 | W130 | W132 |
|  | N7 | W120 | W150 |  | O6 | W147 | W160, W188 |
|  | O6 | W122 | W156 |  | N3 |  | W25* |
|  | N3 | W140 | W105* |  | N2 | W18* | W25* |
|  | N2 | W20, W140 | W11* |  |  |  |  |
